# Meat consumption and risk of breast cancer in the UK Women's Cohort Study

**DOI:** 10.1038/sj.bjc.6603689

**Published:** 2007-04-03

**Authors:** E F Taylor, V J Burley, D C Greenwood, J E Cade

**Affiliations:** 1 Nutritional Epidemiology Group, 30–32 Hyde Terrace, University of Leeds, Leeds, LS2 9LN, UK; 2 Biostatistics Unit, 30–32 Hyde Terrace, University of Leeds, Leeds, LS2 9LN, UK

**Keywords:** prospective studies, breast neoplasms, meat, risk factors

## Abstract

We performed a survival analysis to assess the effect of meat consumption and meat type on the risk of breast cancer in the UK Women's Cohort Study. Between 1995 and 1998 a cohort of 35 372 women was recruited, aged between 35 and 69 years with a wide range of dietary intakes, assessed by a 217-item food frequency questionnaire. Hazard ratios (HRs) were estimated using Cox regression adjusted for known confounders. High consumption of total meat compared with none was associated with premenopausal breast cancer, HR=1.20 (95% CI: 0.86–1.68), and high non-processed meat intake compared with none, HR=1.20 (95% CI: 0.86–1.68). Larger effect sizes were found in postmenopausal women for all meat types, with significant associations with total, processed and red meat consumption. Processed meat showed the strongest HR=1.64 (95% CI: 1.14–2.37) for high consumption compared with none. Women, both pre- and postmenopausal, who consumed the most meat had the highest risk of breast cancer.

Although evidence that links meat consumption with cancers of the stomach, colorectum and pancreas is increasing (Sandhu *et al*, 2001; Gonzalez *et al*, 2006; Larsson *et al*, 2006a, 2006b; Lewin *et al*, 2006), studies of meat consumption and breast cancer have produced more conflicting results. A meta-analysis of 31 case–control and cohort studies published before 2003 found a 17% increase in risk associated with the highest category of meat intakes (Boyd *et al*, 2003). However, a pooled analysis of the raw data from eight prospective cohort studies from North America, Canada and Western Europe was unable to demonstrate such an association (Missmer *et al*, 2002).

Certain evidence suggests an interaction between cooking methods and diet in breast cancer causation. Studies, however, are few and inconsistent. A case–control study of Chinese women in Shanghai found that the positive association with red-meat intake was primarily restricted to those who used deep-frying cooking methods, particularly among those who deep-fried foods to well-done (Dai *et al*, 2002) suggesting an effect of heterocyclic amines or other carcinogens formed at high temperatures. However, the Nurses' Health nested case–control study found no increase in risk with cooking method or meat intake even for consumption of charred meat more than once a week in rapid acetylators (Gertig *et al*, 1999).

Some of the inconsistency in findings may be owing to differences in definitions of total meat, red, and processed meats and in the derivation of the meat content of meat dishes. Other inconsistencies may arise owing to biases, errors and the homogeneity of diet within individual population groups (Hankin, 1993; Kaaks and Riboli, 1997a). The UK Women's Cohort Study (UKWCS), which was established in 1993 to investigate diet in relation to cancer and mortality from selected causes, is well placed to examine meat consumption and breast cancer risk, the subject of this paper.

## MATERIALS AND METHODS

### Study population

The UKWCS, described previously (Cade *et al*, 2004, 2007), was formed from 500 000 responders to a direct mail survey of the World Cancer Research Fund (WCRF) after permission to carry out the baseline study was obtained from 174 local research ethics committees (Woodhouse *et al*, 1997). Seventy-five percent of the responders agreed to take part in a more detailed survey; those eligible for inclusion were women, aged between 35 and 69 years at the completion date of the original mail survey. The 35 372 women who returned completed questionnaires formed the UKWCS, this cohort being specifically designed to have a wide range of dietary intakes and patterns to increase the potential power to detect statistically significant associations between specific diets and disease; 28% are self-reported vegetarians.

Baseline data were gathered between 1995 and 1998 using a 217-item postal food frequency questionnaire (FFQ), developed from that of the European Prospective Investigation into Cancer and Nutrition (EPIC) study (Linseisen *et al*, 2002). This was validated in terms of nutrients, against a semi-weighed 4-day food diary (Spence *et al*, 2002).

Details of women fulfilling the eligibility criteria were submitted to the UK Office of National Statistics and subsequently flagged on the NHS central register. Incident cancers and cause of death were coded according to the International Classification of Diseases 9 and 10. The investigation censor date was 31st October 2004, with median follow-up of 8 years when a total of 1750 incident malignant cancer cases had been recorded, including 283 premenopausal and 395 postmenopausal breast cancers. Menopausal status was based on answers to the baseline questionnaire regarding menstrual and obstetric history and the age at baseline. Power calculations suggested 283 premenopausal breast cancer cases would give approximately 80% power to detect a relative risk of 1.4 comparing two levels of a binary exposure with equal numbers in each group (*P*<0.05), or more than 90% power for a relative risk of 1.5. In terms of postmenopausal breast cancer, 395 cases would give approximately 90% power to detect a relative risk of 1.4 (*P*<0.05). Analysing the exposure as a continuous variable would provide even more power.

### Meat consumption

For the purpose of the study, meat types and meat dishes were grouped into the following categories: red meat, poultry, offal and processed meat. Total meat was the sum of these four categories. Non-processed was the sum of red meat, poultry and offal. Red meat consisted of beef, pork, lamb and other red meats included in mixed dishes, for example, meat lasagne, moussaka, ravioli and filled pasta with sauce; poultry included roast chicken, chicken slices, bread crumbed chicken, chicken or turkey in a creamy sauce and chicken curry; meats considered as processed were bacon, ham, corned beef, spam, luncheon meats, sausages, pies, pasties, sausage rolls, liver pate, salami and meat pizza; offal (organ meats) existed as a single item on the FFQ.

Daily intakes of each of the four main meat types (red, poultry, offal and processed) were calculated by summing the daily intakes of the individual food items within each meat type as described above. Intakes of each item were determined by using the frequency categories to estimate the number of daily portions. These were then converted into weights by referring to standard portion sizes for each food item (Food Standards Agency, 2002). Intakes of each meat type were grouped into consumption categories of ‘none’, ‘low’, ‘medium’ and ‘high’ by classing zero intakes as ‘non-consumers' and dividing non-zero intakes into tertiles. Consumption of offal tended to be more limited and naturally fell into the three categories ‘none’, ‘low’ and ‘high’ consumption only, where low consumption was defined as 2 g or less per day and high as over 2 g per day.

### Statistical analysis

Exposures of interest were total meat consumption, non-processed *vs* processed meat consumption and consumption of different meat types. Processed meat formed a separate category to be compared against non-processed meat. Survival analyses were conducted in Stata version 9 using Cox regression weighted by the inverse of the probability of being sampled to take into account the large proportion of vegetarians in the cohort. The time variable used in the survival analysis was time in the study (person years), calculated as the time from the date the questionnaire was filled in until either a report of incident breast cancer, death or the censor date of the analysis, whichever came first. Women with extremely high or low total energy intake (more than 6000 kcal and less than 500 kcal) were excluded, as were women with prevalent breast cancer.

Two models were developed. Model 1 adjusted only for age (continuous) and energy intake by the residuals method (split into quartiles) (Willett and Stampfer, 1986; Margetts and Nelson, 2000). Model 2 adjusted for age, energy intake, body mass index (BMI) (continuous), physical activity (continuous), parity (no children, 1–2 children, 3–4 children and 5+ children) and combined fruit and vegetable consumption (split into quartiles). Smoking status, hormone replacement therapy use (HRT) and oral contraceptive pill use were also included and all classed as present, past or never. Additional confounders were included such as socioeconomic class (professional and managerial, intermediate, and routine and manual), level of educational qualifications gained (none beyond age 14, O level, A level and degree level). Fractional polynomials were used to fit a smooth curve to the relationship between breast cancer and total meat intake using Model 2.

As breast cancer may represent different diseases in the two menopause status groups, an initial analysis combined both groups, incorporating menopausal status as a confounder in the model. As a test for interaction between meat consumption and menopausal status confirmed a potential modifying effect of menopausal status, we have treated pre- and postmenopausal women independently. The proportional hazards assumption was checked using graphical methods of log–log curves and Schoenfeld goodness of fit tests (Schoenfeld, 1982), which confirmed the hazards were proportional. Owing to the likelihood of differences in lifestyle characteristics between vegetarians and meat eaters in addition to the absence of the meat component within their diet, sensitivity analyses were undertaken excluding vegetarians. The sensitivity of results to excluding women with any cancer incident within 1 year of entry to the study, and to the model building strategy was assessed. Further analysis of sensitivity of results to the menopausal categorisation was carried out by excluding women aged 48–55 years whose menopausal status may have been ambiguous. HRT users (past and present) were also excluded in a sensitivity analysis.

## RESULTS

### Basic characteristics and meat consumption in the cohort

Characteristics of the 33 725 women in the study are shown in Table 1. At baseline, the mean age was 52 years and the average BMI 24.5 kg m^−2^. Cohort participants were relatively health conscious, with low rates of smoking (11%) and alcohol consumption more than once per week (52%). Most did not use full-fat milk (28 383, 88%), and a large proportion reported taking dietary supplements (18 561, 58%). Meat eaters account for a higher percentage of present HRT users than vegetarians, although it must be taken into consideration that vegetarians tend to be younger and therefore less likely to be using HRT. In general, the cohort is well educated and middle class where 8784 (27%) had been educated to degree level and 20 879 (63%) worked in professional or managerial positions. More detail regarding the cohort women has been provided previously (Cade *et al*, 2004).

Table 1 shows that non-meat consumers were younger, more physically active, and had a lower mean BMI than consumers. High meat consumers were more likely to be smokers, had the highest total energy intake, highest mean BMI, highest proportion with no education beyond age 14 and lowest proportion employed in professional or managerial occupations. Medium meat consumers were most likely to be low fruit and vegetable consumers (less than 400 g daily). The lowest energy intake was seen in the group with low meat consumption.

### Meat consumption and breast cancer

The initial analysis combining both pre- and postmenopausal women to test for effect modification by menopausal status (Table 2) showed several significant interactions. Indeed, when independent analyses were conducted for each menopausal status, trends were considerably different.

The associations between meat consumption and premenopausal breast cancer are presented in Table 3 for both Model 1 and Model 2. Use of the complex model showed risk of breast cancer to increase with consumption of total meat, HR (hazard ratios)=1.20 (95% CI: 0.86–1.68) for high consumers *vs* non-consumers. The estimated relative risk for an increase in total meat consumption of 50 g day^−1^ (approximately half a portion) was 1.12 (95% CI: 1.02–1.23, *P*_trend_=0.02). Non-processed meat consumption was positively associated with risk, HR=1.20 (95% CI: 0.86–1.68) for high consumers *vs* non-consumers with a relative risk per 50 g day^−1^ of 1.13 (95% CI: 1.01–1.26, *P*_trend_=0.03). The association with processed meat was not statistically significant although the risk in high consumers was similar to that observed in non-processed meat. The borderline non-significant association with red meat consumption tended to show the largest effect sizes of all meat types, HR=1.32 (95% CI: 0.93–1.88) for high consumption *vs* the reference category with relative risk per 50 g day^−1^ of 1.13 (95% CI: 0.99–1.29, *P*_trend_=0.08).

In postmenopausal women, slight positive trends were observed across the low, medium and high meat categories with a more marked difference between those not consuming meat and those that do. However, splitting the meat categories into more groups by dividing the low consumers into low and very low consumers strengthened the dose response relationship with meat consumption. There was a tendency for the point estimates to be somewhat larger in postmenopausal than in premenopausal women (using Model 2), as shown in Table 4. Total meat intake was positively associated with postmenopausal breast cancer, HR=1.63 (95% CI: 1.10–2.30) for high consumption *vs* the reference category, and when treated as a continuous variable, resulted in a significant linear trend and relative risk per 50 g day^−1^ of 1.10 (95% CI: 1.01–1.20, *P*_trend_ =0.02). Relationships between both processed meat and red meat and postmenopausal breast cancer were also significant. Risks for the three meat types were similar when considering HRs of the categorical analysis, however, fitting meat in the model as a continuous predictor resulted in a much stronger relationship with processed meat, giving a relative risk per 50 g day^−1^ of 1.64 (95% CI: 1.09–2.27, *P*_trend_=0.003).

**Figure 1:**
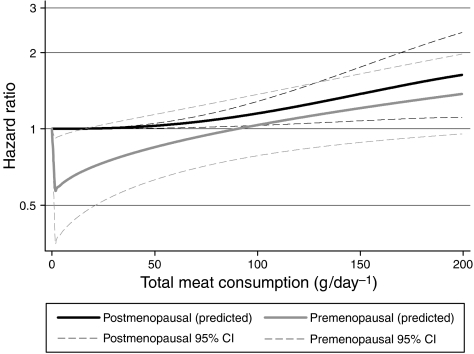
Association between total meat intake and breast cancer for pre- and postmenopausal women.

Hazard ratios in the highest meat consumption category for Model 1 in premenopausal women were slightly lower than for Model 2 for all meat types with the exception of offal (total meat: Model 1 HR=1.16, Model 2 HR=1.20). Tests for trend were more significant in Model 2. The opposite is true for postmenopausal risk where HRs are lowered in the refined model and *P*-values become less significant with greater adjustments. Figure 1 presents the fitted curve from fractional polynomials for total meat intake showing similar increasing risk with increasing total meat intake for both pre- and postmenopausal women, apart from premenopausal women with low meat intake who appear at lower risk than vegetarians.

In the sensitivity results excluding vegetarians, estimates were broadly similar and conclusions unchanged, emphasising a dose response across the consumption categories of meat in both pre- and postmenopausal women. Sensitivity analyses for ambiguous menopausal status and women with cancer within 1 year of entry did not substantially alter HRs or overall trends. The links between meat consumption, cooking methods (grilling, frying and casseroling of meat) and risk were investigated by considering interactions within Model 2; there was no evidence of changes in risk. Excluding HRT users from the analysis of postmenopausal women appeared to strengthen the relationship with breast cancer.

## DISCUSSION

The UKWCS is one of the largest cohorts investigating diet and cancer in women in the UK. It was designed to include participants with a wide range of dietary exposures to optimise comparisons between different levels of meat intake, as proposed previously (Kaaks and Riboli, 1997b; Schatzkin *et al*, 2001). In our analysis, significant increased risks of incident premenopausal breast cancer in relation to increased consumption of total meat and non-processed meat were observed. Borderline non-significant associations with red meat were also seen. We found positive associations between postmenopausal breast cancer and total meat, processed meat and red meat consumption.

Relationships between both pre- and postmenopausal breast cancer and total and red meat consumption confirm findings of a case control study among Chinese women in Shanghai where positive associations were observed in pre- and postmenopausal breast cancers combined (Dai *et al*, 2002). Although pre- and postmenopausal women were also considered separately, the full data were not shown. Positive associations among those who usually deep-fried red meat until well done, were found in both groups, although statistically significant only in premenopausal women.

The association with red meat intake and both pre- and postmenopausal breast cancer may be due to a combination of nutritionally related factors, such as content of fat, protein and iron, and/or meat preparation (eg cooking or preserving methods) (Sinha, 2002). A comparison of high consumer HRs for all meat types investigated showed that high consumers of red meat are most at risk of premenopausal breast cancer when compared with non-consumers (HR=1.32, 95% CI: 0.93–1.88). The association found between non-processed meat (red meat, poultry and offal) could also be caused by the red meat component within the non-processed meat category.

Results of a large case–control study (10 149 cases and 7990 controls) in northern Italy between 1983 and 1996 also found significant positive associations of breast cancer (combined analysis of pre- and post menopausal women) with red meat consumption (Tavani *et al*, 2000). In addition, a meta-analysis of 12 case–control and five cohort studies published between 1966 and 1993 found increased risks of breast cancer (combined pre- and postmenopausal) in high consumers, the association with red meat (RR=1.54, 95% CI: 1.31–1.82) being stronger than that observed for total meat (Boyd *et al*, 2003). However, a pooled analysis of eight previous cohort studies has shown no significant association between consumption of total meat, red meat or white meat and risk of breast cancer (Missmer *et al*, 2002) in both combined and separate analyses of pre- and postmenopausal women. The pooled analysis was not able to correct for measurement error and there were considerable differences in questionnaire design between studies limiting the power of specific food analyses. Also, meat-cooking practices could not be taken into account.

Previous studies have tended to find inverse relationships with consumption of poultry (Delfino *et al*, 2000; Ronco *et al*, 2003) and have generally been statistically non-significant. Our findings do not provide strong evidence of an association with poultry intake and breast cancer in either pre- or postmenopausal women. However, another study showed statistically significant inverse trends between consumption of poultry and postmenopausal breast cancer (Ambrosone *et al*, 1998). One study found that risks were increased when chicken was consumed with skin suggesting that fat rather than muscle meat may be the cause (Ronco *et al*, 2003). Other studies have suggested a link between fat and breast cancer (Howe *et al*, 1991; Willett *et al*, 1992; Hunter *et al*, 1996; Smith-Warner *et al*, 2001; Boyd *et al*, 2003; Cho *et al*, 2003).

Although HRs for pre menopausal breast cancer indicate a positive association with meat intake, low consumers are at less risk than vegetarians. Low meat consumers also had the lowest energy and fat intakes, but including the percentage of energy from fat as a confounder and also calculated using the residuals method (Willett and Stampfer, 1986) did not significantly modify the risk estimates. Vegetarians possess other characteristics other than not consuming meat and these may influence the association with risk in some way. Although we adjusted for characteristics known to be represented differently in meat eaters and vegetarians (Davey *et al*, 2003; Cade *et al*, 2004) and performed various sensitivity analyses with the exclusion of the vegetarian group, some residual confounding may remain.

Genetic factors only account for a small proportion of breast cancers (approximately 5–10%). The UKWCS are expected to have a higher proportion than this as family history of breast cancer may have encouraged them to become WCRF supporters. In addition, some may have taken up a vegetarian diet in the belief that it is protective against breast cancer. However, if these women are also genetically predisposed to breast cancer, then the chances of developing breast cancer are increased. This is more likely among the premenopausal women because genetic causes tend to lead to early onset of breast cancer. This could explain why, in the premenopausal women, vegetarians have a higher risk than others.

Risks for pre- and postmenopausal women were examined separately, based on variability in some risk factors and because breast cancer may represent different diseases in these groups (Ambrosone *et al*, 1998). Also, mean intakes of certain meats were found to differ significantly between the two menopausal groups. In addition, after the menopause, increased deposition of adipose tissue, the major site for oestrogen synthesis, will tend to elevate the level of endogenous oestrogens (Siiteri, 1987). The association between intake of carcinogens from foods cooked at high temperature and breast cancer risk may be modified by oestrogens and oestrogen-related factors. Other analysis has found a difference in impact of dietary fibre on risk of breast cancer between pre- and postmenopausal women (Cade *et al*, 2007).

There are several mechanisms whereby meat intake may contribute to breast cancer risk. Meat and in particular processed meats can be a rich source of saturated fat. Although effect on mammary carcinogenesis has been shown in animals, its human relevance is controversial (Ip, 1993). A review of prospective studies has shown that dietary fat reduction can lower serum oestradiol levels (Wu *et al*, 1999). Many established risk factors are linked to oestrogens such as early menarche, late menopause and obesity in postmenopausal women (Key and Verkasalo, 1999). Other mechanisms related to the formation of heterocyclic amines during cooking or nitroso compounds found in processed meat (Willett, 2005) may be altered by inherited polymorphisms such as the rapid variant of *N*-acetyltransferase 2 (Williamson *et al*, 2005). Red meat also contains high biological-value protein and important micronutrients, all of which are essential for good health throughout life.

In postmenopausal women, the largest effects were with processed meat and this was statistically significant, HR=1.64 (95% CI: 1.14–2.37) for high *vs* non-consumers with relative risk per 50 g day^−1^ of 1.64 (95% CI: 1.19–2.27, *P*_trend_=0.003). Risks were increased by almost 50% for even low consumers of processed meat. A case–control study in a subcohort of the Nurses' Health Study (466 cases) supports this, breast cancer (combined pre- and postmenopausal) being 40% more likely in women consuming more than 0.07 portions of bacon daily in comparison with non-consumers (Gertig *et al*, 1999). Although trends were statistically non-significant, non-processed meat and poultry were both positively associated with postmenopausal breast cancer. Differences in outcome trends for pre- and postmenopausal women may be owing to the fact that oestrogen metabolism pathways differ according to menopausal status (Muti *et al*, 2000). If meat influences breast cancer by affecting oestrogen metabolism, the effect may be relatively more important among women with lower levels of circulating oestrogens.

The strength of this study was the wide range of meat intake within the cohort which reduces measurement error (White *et al*, 1994; Kaaks and Riboli, 1997b; Schatzkin *et al*, 2001). Previous studies have been limited in terms of the FFQs used which may not have been designed to capture specific food groups in sufficient detail (Missmer *et al*, 2002). An analysis of EPIC-Norfolk data concluded that dietary measurement error through the use of their FFQ may explain the absence of a significant association with dietary fat and breast cancer risk as well as some of the previously reported inconsistencies on meat (Bingham *et al*, 2003).

In conclusion, women generally consuming most total meat, red and processed meat were at the highest increased risk compared with non-meat consumers, though red and processed meat were only statistically significant postmenopausally. Effect sizes were smaller in non-processed meat and only statistically significant in premenopausal women. There were no statistically significant linear associations with consumption of poultry or offal in either pre- or postmenopausal women. This study indicates relationships with certain meats and breast cancer in both pre- and postmenopausal women and merits further investigation in a larger study.

## Figures and Tables

**Table 1:** Baseline characteristics by category of meat consumption

	**Total meat consumption**				
	**None (0 ***g***) *n*=8881**	**Low (<62 ***g***) *n*=8281**	**Medium (62–103 ***g***) *n*=8282**	**High (>103 ***g***) *n*=8281**	**Total *n*=33 725**
Age (years), mean (s.d.)	49 (8)	53 (9)	54 (9)	53 (9)	52 (9)
BMI (kg m^−2^), mean (s.d.)	23.3 (3.8)	24.0 (4.1)	24.9 (4.3)	25.7 (5.9)	24.5 (4.4)
Energy intake (MJ), mean (s.d.)	9.8 (3.0)	8.9 (2.8)	9.4 (2.5)	11.2 (3.1)	9.9 (3.4)
Physical activity (min), mean (s.d.)	17 (29)	14 (28)	13 (26)	14 (31)	14 (28)
Current smoker (%)	10	11	10	13	11
Current HRT use (%)	14	20	22	23	20
Current OCP use (%)	5	4	3	4	4
No children (%)	27	23	17	14	20
Professional and managerial (%)	70	66	60	57	63
Low intake of fruit and vegetables (%)	18	27	31	25	25
Total meat (grams), mean (s.d.)	0	34 (19)	82 (12)	148 (48)	65 (62)

BMI=body mass index; HRT=hormone replacement therapy.

**Table 2:** Combined pre- and postmenopausal breast cancer

				**Model 1^a^**		**Model 2^b^**	
	**Consumption (***g***/day)**	**Person years (mean (s.d.))**	**Cases/non-cases**	**HR**	**95% CI**	**HR**	**95% CI**
*Total meat*							
*Categorical*							
None (ref)	0	7.50 (0.68)	149/8881	1.00	—	1.00	—
Low	<62	7.25 (0.92)	162/8281	1.10	(0.88, 1.39)	1.04	(0.82, 1.33)
Medium	62–103	6.79 (0.98)	182/8282	1.30	(1.04, 1.63)	1.25	(0.98, 1.60)
High	>103	6.63 (0.94)	185/8281	1.40	(1.12, 1.75)	1.34	(1.05, 1.71)
							
Continuous			Risk per 50 g/day	1.11	(1.05, 1.17)	1.11	(1.04, 1.18)
				*P* (trend) <0.001		*P* (trend)=0.001	
Test for effect modification by menopausal status				0.0269		0.0492	
							
*Non-processed meat (including red meat, poultry and offal)*							
*Categorical*							
None (ref)	0	7.51 (0.67)	151/9135	1.00	—	1.00	—
Low	<50	7.22 (0.93)	163/8196	1.12	(0.89, 1.41)	1.07	(0.84, 1.36)
Medium	50–84	6.79 (0.98)	185/8198	1.34	(1.07, 1.68)	1.34	(1.05, 1.70)
High	>84	6.63 (0.94)	179/8196	1.37	(1.09, 1.72)	1.33	(1.04, 1.69)
							
Continuous			Risk per 50 g/day	1.11	(1.04, 1.18)	1.10	(1.03, 1.19)
				*P* (trend)=0.003		*P* (trend)=0.007	
Test for effect modification by menopausal status				0.0454		0.0452	
							
*Processed meat*							
*Categorical*							
None (ref)	0	7.51 (0.70)	175/10306	1.00	—	1.00	—
Low	<10	7.08 (0.94)	160/7824	1.17	(0.93, 1.47)	1.19	(0.94, 1.53)
Medium	10–20	6.77 (0.99)	172/7814	1.31	(1.04, 1.64)	1.30	(1.02, 1.66)
High	>20	6.69 (0.95)	171/7781	1.35	(1.08, 1.70)	1.39	(1.09, 1.78)
							
Continuous			Risk per 50 g/day	1.40	(1.18, 1.67)	1.59	(1.22, 2.06)
				*P* (trend) <0.001		*P* (trend) <0.001	
Test for effect modification by menopausal status				0.1365		0.4523	
							
*Red meat*							
*Categorical*							
None (ref)	0	7.52 (0.68)	186/11199	1.00	—	1.00	—
Low	<32	7.15 (0.97)	162/7512	1.28	(1.02, 1.61)	1.21	(0.95, 1.54)
Medium	32–57	6.72 (0.95)	163/7560	1.36	(1.08, 1.71)	1.40	(1.10, 1.78)
High	>57	6.57 (0.93)	167/7454	1.47	(1.17, 1.84)	1.41	(1.11, 1.81)
							
Continuous			Risk per 50 g/day	1.12	(1.03, 1.21)	1.12	(1.03, 1.22)
				*P* (trend)=0.005		*P* (trend)=0.007	
Test for effect modification by menopausal status				0.0325		0.0577	
							
*Poultry*							
*Categorical*							
None (ref)	0	7.48 (0.70)	160/9607	1.00	—	1.00	—
Low	<14	6.96 (0.98)	160/7401	1.24	(0.97, 1.57)	1.19	(0.92, 1.54)
Medium	14–23	6.81 (0.99)	191/8678	1.30	(1.03, 1.63)	1.25	(0.98, 1.59)
High	>23	6.88 (0.97)	167/8039	1.25	(0.99, 1.58)	1.22	(0.95, 1.56)
							
Continuous			Risk per 50 g/day	1.14	(0.95, 1.35)	1.11	(0.92, 1.34)
				*P* (trend)=0.154		*P* (trend)=0.285	
Test for effect modification by menopausal status				0.7242		0.8897	
							
*Offal*							
*Categorical*							
None (ref)	0	7.23 (0.88)	366/20499	1.00	—	1.00	—
Low	⩽2	6.79 (0.99)	190/7833	1.34	(1.11, 1.61)	1.35	(1.11, 1.64)
—	—			—	—	—	—
High	>2	6.73 (0.99)	122/5393	1.22	(0.99, 1.52)	1.17	(0.93, 1.48)
							
Continuous			Risk per 50 g/day	1.92	(0.81, 4.53)	1.75	(0.68, 4.50)
				*P* (trend)=0.136		*P* (trend)=0.248	
Test for effect modification by menopausal status				0.6334		0.6039	

CI=confidence intervals; HR=hazard ratios.

a Adjusting for age, energy intake and menopausal status.

b Adjusting for age, energy intake, menopausal status, BMI, physical activity, smoking status, HRT use, OCP use, parity, total fruit and vegetable intake.

**Table 3:** Pre menopausal breast cancer

				**Model 1^a^**		**Model 2^b^**	
	**Consumption (***g***/day)**	**Person years (mean (s.d.))**	**Cases/non-cases**	**HR**	**95% CI**	**HR**	**95% CI**
*Total meat*							
*Categorical*							
None (ref)	0	7.50 (0.66)	98/5435	1.00	—	1.00	—
Low	<62	7.35 (0.85)	52/3586	0.72	(0.51, 1.03)	0.68	(0.47, 0.99)
Medium	62–103	6.96 (0.99)	63/3309	1.00	(0.72, 1.39)	1.08	(0.76, 1.53)
High	>103	6.83 (0.97)	70/3334	1.16	(0.85, 1.58)	1.20	(0.86, 1.68)
Continuous			Risk per 50 g/day	1.10	(1.00,1.20)	1.12	(1.02, 1.23)
				*P* (trend)=0.046		*P* (trend)=0.02	
							
*Non-processed meat (including red meat, poultry and offal)*							
*Categorical*							
None (ref)	0	7.50 (0.66)	98/5556	1.00	—	1.00	—
Low	<50	7.32 (0.86)	51/3539	0.73	(0.51, 1.04)	0.69	(0.47, 1.01)
Medium	50–84	6.97 (1.01)	66/3271	1.09	(0.79, 1.51)	1.18	(0.83, 1.66)
High	>84	6.83 (0.97)	68/3298	1.17	(0.86, 1.6)	1.20	(0.86, 1.68)
Continuous			Risk per 50 g/day	1.10	(0.99, 1.22)	1.13	(1.01, 1.26)
				*P* (trend)=0.069		*P* (trend)=0.03	
							
*Processed meat*							
*Categorical*							
None (ref)	0	7.51 (0.68)	109/6069	1.00	—	1.00	—
Low	<10	7.22 (0.89)	55/3196	0.88	(0.62, 1.24)	0.94	(0.65, 1.36)
Medium	10–20	6.95 (1.00)	56/3223	0.94	(0.67, 1.32)	1.04	(0.72, 1.51)
High	>20	6.89 (0.97)	63/3176	1.13	(0.82, 1.56)	1.20	(0.85, 1.7)
Continuous			Risk per 50 g/day	1.44	(0.96, 2.18)	1.45	(0.95, 2.23)
				*P* (trend)=0.079		*P* (trend)=0.09	
							
*Red meat*							
*Categorical*							
None (ref)	0	7.52 (0.66)	113/6463	1.00	—	1.00	—
Low	<32	7.24 (0.90)	50/3328	0.83	(0.58, 1.18)	0.80	(0.55, 1.17)
Medium	32–57	6.91 (0.98)	59/3050	1.11	(0.79, 1.55)	1.19	(0.83, 1.7)
High	>57	6.78 (0.98)	61/2823	1.28	(0.93, 1.77)	1.32	(0.93, 1.88)
Continuous			Risk per 50 g/day	1.10	(0.97, 1.25)	1.13	(0.99, 1.29)
				*P* (trend)=0.143		*P* (trend)=0.08	
							
*Poultry*							
*Categorical*							
None (ref)	0	7.50 (0.67)	99/5700	1.00	—	1.00	—
Low	<14	7.17 (0.96)	53/2854	1.05	(0.74, 1.48)	1.07	(0.74, 1.54)
Medium	14–23	6.99 (0.99)	64/3486	1.06	(0.77, 1.47)	1.05	(0.75, 1.49)
High	>23	7.00 (0.95)	67/3624	1.10	(0.81, 1.51)	1.15	(0.82, 1.61)
Continuous			Risk per 50 g/day	1.23	(0.91, 1.65)	1.28	(0.93, 1.75)
				*P* (trend)=0.172		*P* (trend)=0.13	
							
*Offal*							
*Categorical*							
None (ref)	0	7.32 (0.82)	183/10616	1.00	—	1.00	—
Low	⩽2	6.97 (0.98)	69/3252	1.24	(0.93, 1.66)	1.32	(0.98, 1.78)
—	—			—	—	—	—
High	>2	6.96 (1.00)	31/1796	0.99	(0.67, 1.47)	0.96	(0.63, 1.45)
Continuous			Risk per 50 g/day	1.53	(0.22, 10.36)	1.63	(0.22, 11.9)
				*P* (trend)=0.665		*P* (trend)=0.63	

CI=confidence intervals; HR=hazard ratios.

a Adjusting for age and energy intake.

b Adjusting for age, energy intake, BMI, physical activity, smoking status, HRT use, OCP use, parity, total fruit and vegetable intake.

**Table 4:** Postmenopausal breast cancer

				**Model 1^a^**		**Model 2^b^**	
	**Consumption (***g*** per day)**	**Person years (mean(s.d.))**	**Cases/non-cases**	**HR**	**95% CI**	**HR**	**95% CI**
*Total meat*							
*Categorical*							
None (ref)	0	7.51 (0.70)	51/3297	1.00		1.00	—
Low	<62	7.18 (0.96)	110/4533	1.68	(1.19, 2.36)	1.52	(1.06, 2.18)
Medium	62–103	6.66 (0.95)	119/4791	1.81	(1.29, 2.56)	1.58	(1.09, 2.27)
High	>103	6.48 (0.88)	115/4762	1.87	(1.33, 2.63)	1.63	(1.13, 2.35)
Continuous			Risk per 50 g/day	1.11	(1.03, 1.19)	1.10	(1.01, 1.20)
				*P* (trend)=0.004		*P* (trend)=0.021	
							
*Non-processed meat (including red meat, poultry and offal)*							
*Categorical*							
None (ref)	0	7.51 (0.70)	53/3428	1.00		1.00	—
Low	<50	7.14 (0.98)	112/4494	1.62	(1.14, 2.31)	1.53	(1.06, 2.21)
Medium	50–84	6.67 (0.94)	119/4742	1.74	(1.22, 2.46)	1.63	(1.13, 2.36)
High	>84	6.49 (0.89)	111/4719	1.72	(1.21, 2.44)	1.59	(1.1, 2.3)
Continuous			Risk per 50 g/day	1.11	(1.01, 1.21)	1.09	(0.99, 1.20)
				*P* (trend)=0.023		*P* (trend)=0.088	
							
*Processed meat*							
*Categorical*							
None (ref)	0	7.51 (0.72)	66/4062	1.00		1.00	—
Low	<10	6.99 (0.97)	105/4468	1.53	(1.09, 2.15)	1.48	(1.04, 2.12)
Medium	10–20	6.64 (0.96)	116/4419	1.76	(1.26, 2.47)	1.60	(1.12, 2.29)
High	>20	6.54 (0.91)	108/4434	1.70	(1.21, 2.39)	1.64	(1.14, 2.37)
Continuous			Risk per 50 g/day	1.40	(1.16, 1.70)	1.64	(1.19, 2.27)
				*P* (trend)=0.001		*P* (trend)=0.003	
							
*Red meat*							
*Categorical*							
None (ref)	0	7.52 (0.70)	73/4550	1.00		1.00	—
Low	<32	7.08 (1.01)	112/4022	1.78	(1.28, 2.47)	1.63	(1.15, 2.31)
Medium	32–57	6.59 (0.90)	104/4347	1.67	(1.19, 2.33)	1.64	(1.15, 2.34)
High	>57	6.44 (0.87)	106/4464	1.73	(1.24, 2.41)	1.56	(1.09, 2.23)
Continuous			Risk per 50 g/day	1.13	(1.02, 1.25)	1.12	(1.01, 1.26)
				*P* (trend)=0.019		*P* (trend)=0.040	
							
*Poultry*							
*Categorical*							
None (ref)	0	7.45 (0.76)	61/3747	1.00		1.00	—
Low	<14	6.82 (0.97)	107/4387	1.43	(1, 2.05)	1.32	(0.9, 1.93)
Medium	14–23	6.70 (0.97)	127/5001	1.51	(1.07, 2.14)	1.39	(0.96, 2.02)
High	>23	6.78 (0.97)	100/4248	1.41	(0.99, 2.01)	1.30	(0.89, 1.89)
Continuous			Risk per 50 g/day	1.06	(0.85, 1.33)	1.00	(0.78, 1.28)
				*P* (trend)=0.585		*P* (trend)=0.985	
							
*Offal*							
*Categorical*							
None (ref)	0	7.13 (0.92)	183/9517	1.00		1.00	—
Low	⩽2	6.67 (0.97)	121/4391	1.39	(1.09, 1.76)	1.37	(1.05, 1.77)
—	—					—	—
High	>2	6.61 (0.96)	91/3475	1.34	(1.03, 1.73)	1.26	(0.95, 1.67)
Continuous			Risk per 50 g/day	2.01	(0.79, 5.13)	1.62	(0.57, 4.59)
				*P* (trend)=0.142		*P* (trend)=0.363	

CI=confidence intervals; HR=hazard ratios.

a Adjusting for age and energy intake.

b Adjusting for age, energy intake, BMI, physical activity, smoking status, HRT use, OCP use, parity, total fruit and vegetable intake.
